# A Non-Human Primate Model of Severe Pneumococcal Pneumonia

**DOI:** 10.1371/journal.pone.0166092

**Published:** 2016-11-17

**Authors:** Luis F. Reyes, Marcos I. Restrepo, Cecilia A. Hinojosa, Nilam J. Soni, Anukul T. Shenoy, Ryan P. Gilley, Norberto Gonzalez-Juarbe, Julio R. Noda, Vicki T. Winter, Melissa A. de la Garza, Robert E. Shade, Jacqueline J. Coalson, Luis D. Giavedoni, Antonio Anzueto, Carlos J. Orihuela

**Affiliations:** 1 Division of Pulmonary Diseases & Critical Care Medicine, The University of Texas Health Science Center at San Antonio, San Antonio, TX, United States of America; 2 Division of Pulmonary Diseases & Critical Care Medicine, South Texas Veterans Health Care System, San Antonio, TX, United States of America; 3 Department of Microbiology and Immunology, The University of Texas Health Science Center at San Antonio, San Antonio, TX, United States of America; 4 Department of Microbiology, The University of Alabama at Birmingham, Birmingham, AL, United States of America; 5 Department of Pathology, The University of Texas Health Science Center at San Antonio, San Antonio, TX, United States of America; 6 Texas Biomedical Research Institute, San Antonio, TX, United States of America; Louisiana State University, UNITED STATES

## Abstract

**Rationale:**

*Streptococcus pneumoniae* is the leading cause of community-acquired pneumonia and infectious death in adults worldwide. A non-human primate model is needed to study the molecular mechanisms that underlie the development of severe pneumonia, identify diagnostic tools, explore potential therapeutic targets, and test clinical interventions during pneumococcal pneumonia.

**Objective:**

To develop a non-human primate model of pneumococcal pneumonia.

**Methods:**

Seven adult baboons (*Papio cynocephalus*) were surgically tethered to a continuous monitoring system that recorded heart rate, temperature, and electrocardiography. Animals were inoculated with 10^9^ colony-forming units of *S*. *pneumoniae* using bronchoscopy. Three baboons were rescued with intravenous ampicillin therapy. Pneumonia was diagnosed using lung ultrasonography and *ex vivo* confirmation by histopathology and immunodetection of pneumococcal capsule. Organ failure, using serum biomarkers and quantification of bacteremia, was assessed daily.

**Results:**

Challenged animals developed signs and symptoms of pneumonia 4 days after infection. Infection was characterized by the presence of cough, tachypnea, dyspnea, tachycardia and fever. All animals developed leukocytosis and bacteremia 24 hours after infection. A severe inflammatory reaction was detected by elevation of serum cytokines, including Interleukin (IL)1Ra, IL-6, and IL-8, after infection. Lung ultrasonography precisely detected the lobes with pneumonia that were later confirmed by pathological analysis. Lung pathology positively correlated with disease severity. Antimicrobial therapy rapidly reversed symptomology and reduced serum cytokines.

**Conclusions:**

We have developed a novel animal model for severe pneumococcal pneumonia that mimics the clinical presentation, inflammatory response, and infection kinetics seen in humans. This is a novel model to test vaccines and treatments, measure biomarkers to diagnose pneumonia, and predict outcomes.

## Introduction

Community-acquired pneumonia (CAP) is the leading cause of infectious death worldwide [1]. In the United States, more than 3.5 million adults develop CAP annually resulting in 10 million hospital visits [2]; with costs in the U.S. alone exceeding 10 billion dollars per year [3]. Of CAP patients that require hospitalization, as many as 22% also require admission to an intensive care unit (ICU) due to the severity of the infection [4]. *Streptococcus pneumoniae* (the pneumococcus), a Gram-positive bacteria, is the most frequent pathogen isolated from CAP patients, responsible for up to 45% of cases [3, 5]. Pneumococcal pneumonia is associated with 10–15% mortality in adults, but can be as high as 40% in elderly patients [6]. Case-fatality rates due to pneumococcal pneumonia have remained unchanged during the past 20 years despite widespread use of appropriate antibiotic treatment and new guidelines recommending pneumococcal vaccination [7].

Historical descriptions of patients with pneumococcal pneumonia during the pre-antibiotic era described that humans developed various degrees of disease severity with a broad array of symptoms and signs, including bacteremia and development of organ failure [8, 9]. Accordingly, some patients were able to overcome infection without antibiotic treatment while others succumbed to severe pneumonia and its complications [9]. Currently, antimicrobial therapy is the cornerstone of pneumonia treatment [2]. Growing efforts are now focused on immune modulation to dampen the detrimental host response to bacterial products that are released when bacteria are destroyed by antimicrobials [7, 10].

Animal models have long been used to characterize and study the molecular basis of pneumococcal disease [11]. Because of its low cost, ease of use, and genetic tractability, the mouse model is the most common animal model used to study pneumococcal disease. Others, such as rats, pigs, and chinchillas, have also been used but far less frequently [11]. The concern with any of these animal models is their translation to human disease. Recently, the ENCODE consortium described that environmental factors play a key role in the evolution of species, resulting in fundamental differences in how humans and mice respond to infections [12, 13]. One key example being that mice are intrinsically resistant to the major *S*. *pneumoniae* toxin pneumolysin [14]. Moreover, humans and small animals differ significantly in respiratory anatomy and physiology [11]. Therefore, common animal models, particularly rodents, do not completely mimic pneumococcal disease in humans. Given these facts, a more relevant animal model is needed to study biological mechanisms, therapeutic targets, and preventive strategies for pneumococcal disease in humans. Along such lines, non-human primates have been successfully used to study highly complicated immunological interactions in organ transplantation experiments [15]. We hypothesize that non-human primates, our closest relatives anatomically and physiologically, will mount a host-pathogen interaction during pneumococcal pneumonia that is most analogous to humans.

Herein we present a non-human primate model of pneumococcal pneumonia using baboons (*Papio cynocephalus*) with and without antimicrobial therapy. We also use and validate lung ultrasonography as an alternative diagnostic tool in baboons with pneumococcal disease. We propose this non-human primate model will help elucidate host-pathogen interactions during pneumococcal disease and serve as a method for studying diagnostic and prognostic biomarkers, therapeutic interventions, and preventive strategies.

## Materials and Methods

This was an experimental study of severe pneumonia in non-human primates after intrabronchial inoculation with *S*. *pneumoniae*.

### Ethics Statement

Studies were performed at the Texas Biomedical Research Institute (TBRI) in San Antonio, Texas. All animal procedures and protocols performed were approved by the Institutional Animal Care and Use Committee (IACUC Number 1443PC6) at the TBRI.

### General Welfare of the Non-Human Primates

For tethered animals, the ceilings of the cages were modified to accommodate the coil of the tether system. Cages are 12 ft^2^. They were on automatic 12hr light/dark cycles (7am/7pm). During the period of time that observations and medications had to be increased into the overnight periods, the minimal lighting necessary was used throughout the experiment. Baboons were given unlimited access to water with an automatic watering system; this was checked for patency daily. Baboons were given biscuits in the mornings and fruit and vegetable enrichment in the afternoons. Consumption was recorded to help assess appetite and general wellness. Additionally, they were offered extra supplements high in protein. Animals were all supplied with manipulable enrichment both inside and hanging on their cages. They were provided auditory stimulus with radios and televisions and human interaction enrichment with the staff throughout the day.

### Animal Preparation

Unrelated adult baboons (*Papio cynocephalus)* were screened to ensure the absence of underlying comorbidities, such as diabetes, hypertension, heart failure, renal failure, or other major conditions before study enrollment. A full chart review and complete physical exam was performed by a licensed veterinarian (MDG) to confirm the healthy status of each animal. Laboratory analyses performed at the time of initial evaluation included blood chemistries (glucose, creatinine, urea, electrolytes), troponin, complete blood cell count (CBC), coagulation profile, liver function tests, and albumin. In addition, a bedside ultrasound examination was performed that included a transthoracic echocardiogram along with lung and renal assessment. Point-of-care lung ultrasound examination followed the BLUE protocol was performed with a portable ultrasound machine (General Electric Logiq E Vet) equipped with a microconvex (GE model 8C-RS, 4.0–10.0 MHz) and a phased-array transducer (GE model 3S-RS, 1.7–4.0 MHz) [16–18].

Animals determined to have a normal baseline health status were enrolled in the study. A continuous monitoring system was surgically implanted to allow real-time recording of heart rate, subcutaneous temperature, and a 3-lead electrocardiogram (ECG) [19]. A sterile triple-lumen central venous catheter was surgically implanted in the right or left femoral vein to facilitate daily blood draws for laboratory monitoring throughout the study. All wires, catheters, and blood sampling ports required for continuous monitoring were protected in a vest that the animals wore throughout the experiment. Animals were allowed to recover for 2 weeks after implantation of the monitoring equipment. During the first 7 days of recovery they received cefazolin 25mg/kg IV every 12 hours to prevent skin and soft tissue infection. For more detailed information, review S1 Text.

### Baseline Procedures

A ***baseline assessment*** was performed on the day of *S*. *pneumoniae* infection prior to inoculation. Animals were sedated with a single intramuscular dose of ketamine at 10mg/kg. Once sedation was achieved, this assessment included measurement of vital signs, collection of blood for laboratory tests and bacterial culture, acquisition of a 12-lead ECG, and performance of a bronchoalveolar lavage (BAL). Whole blood was serially diluted, plated, and incubated overnight at 37°C for assessment of bacterial load [20]. Serum samples were processed for blood chemistries using standard methods [21]. Cytokine and chemokine analyses were performed using a validated 23-panel luminex multiplex assay for non-human primates (see full list of cytokines/chemokines in S1 Table) [22]. A baseline BAL was performed bronchoscopically (Olympus BF, model XP60) with the administration of two 20mL aliquots of sterile saline solution (0.9%NaCl) in the right middle lobe. Recovered aspirate BAL fluid was collected in a sterile container and submitted for laboratory analysis that included bacterial culture as described above.

**Fig 1:**
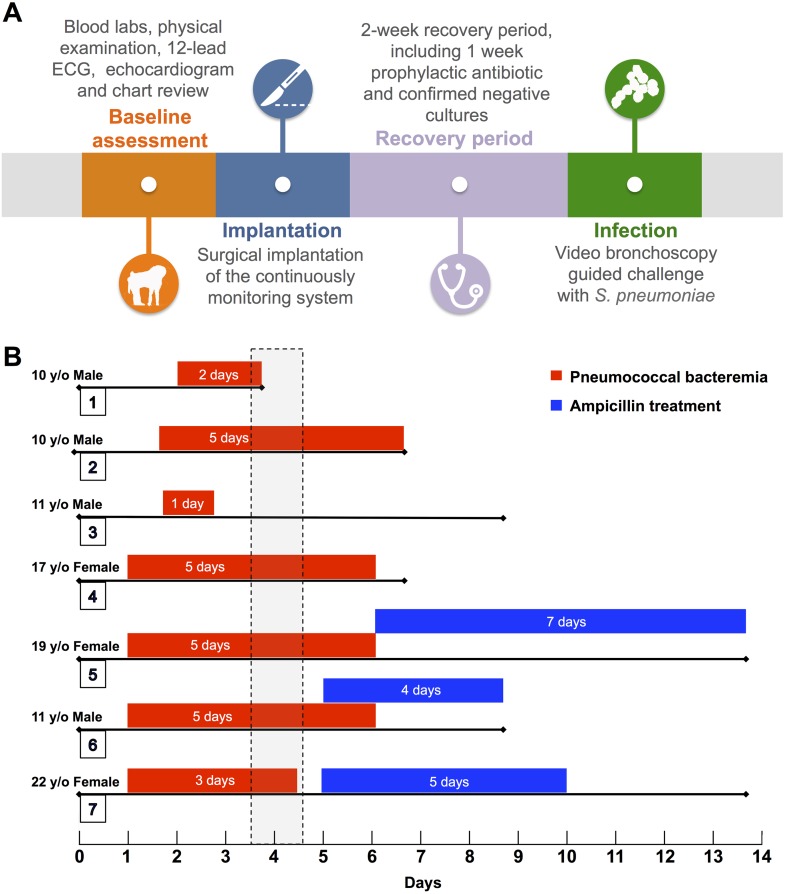
Experiment Overview. (A) Description of the baseline assessment, implantation, recovery period, and infection. (B) Graphical representation of each experiment following intrabronchial challenge with *S*. *pneumoniae*, showing demographics, days with positive blood culture (red bars), onset and duration of antimicrobial therapy (blue bars), and total days of each experiment. Vertical dashed rectangle indicates the time period when a diagnosis of pneumonia was established.

*S*. *pneumoniae* serotype 4 (strain TIGR4) was grown in Todd Hewitt broth at 37°C and 5% CO_2_ to an OD_620_ = 0.5 (mid-logarithmic phase growth; corresponds to 10^8^ colony forming units per mL). The bacterial suspension (10 ml) was centrifuged, washed with sterile phosphate-buffered saline (PBS), and bacteria suspended in 1 mL PBS. Anesthetized baboons were bronchoscopically inoculated with this suspension in the right middle lobe thereby receiving 10^9^ colony-forming units. A general timeline for these procedures is presented in Fig 1A.

### Experiment Follow-Up

Veterinarians performed daily clinical assessments of all animals. Continuous, 24-hour monitoring of temperature, heart rate and ECG were recorded throughout the experiment. Blood collection for CBC, chemistries, cytokine analyses and bacterial load were performed daily at 7:00 a.m. The first animal was used to define the ***diagnosis of pneumonia*** after euthanasia 5 days post-inoculation. Histopathology, lung ultrasound findings, and blood work showing a systemic inflammatory response were used to establish the presence of pneumonia. Bacteremia was defined by positive blood cultures with at least 10^3^ CFU/mL. At day 5 post-infection, three animals received intravenous (IV) administration of ampicillin at a dose of 80 mg/kg/day for 4–5 days before the end of the experiment.

***End of experiment*** procedures included a 12-lead ECG, lung ultrasonography, blood collection for CBC, chemistries, cytokine analyses, and bacterial load. Animals were euthanized with a single IV dose of Fatal-Plus^®^ (pentobarbital sodium) of 10ml/10lbs of body weight. A comprehensive *ex vivo* pathological examination included macroscopic and microscopic evaluation of bilateral lung parenchyma. Hematoxylin and eosin (H&E) stained lung sections from different lobes were scanned with the Aperio Scanscope XT (Aperio, Vista CA) and digital images of the entire tissue sections were created for analysis. Lung tissue bacterial burden was assessed for CFU/g of homogenized tissue. Frozen lung sections were prepared and processed for immunofluorescent microscopy using standard methods [23]. The primary antibody used was rabbit anti-serotype 4 capsular polysaccharide antibody (Statens serum Institut) diluted at 1:1000. The secondary antibody used was fluorescein isothiocyanate (FITC)-labeled goat anti rabbit antibody (Jackson Immuno Research) diluted at 1:1000. For further information about materials and method, review S1 Text.

### Statistical Analysis

All data are presented as medians with interquartile ranges (IQR), or means with standard deviations (SD) as appropriate. Nonparametric Mann-Whitney U tests or two-tailed unpaired Student’s *t*-test were used to compare data at different time points (baseline, pneumonia, and end-of-experiment) of animals treated with or without antibiotics. All statistical calculations were done using Prism 5 software (GraphPad Software: La Jolla, CA). *P* values <0.05 were considered to be significant.

## Results

**Fig 2:**
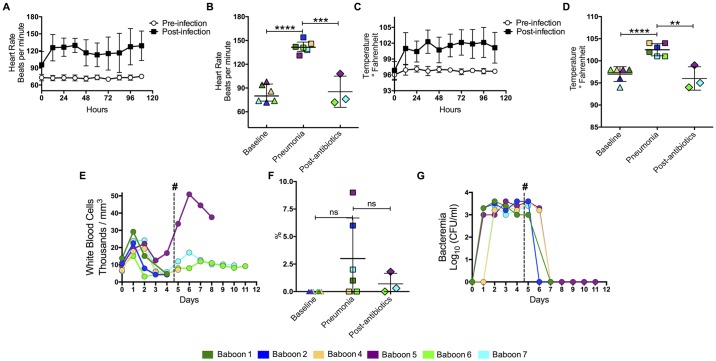
Animals developed systemic inflammatory response syndrome after intrabronchial challenge. Tethered animals were continuously monitored to measure heart rate and temperature. (A) Mean heart rate (n = 6) 5 days before and after intratracheal challenge with *S*. *pneumoniae*. (B) Individual heart rates for the baboons at day 0 (Baseline), day 4 post-infection (Pneumonia), and 4–5 days after antimicrobial treatment (post-antibiotics). Each different colored symbol represents an individual animal (legend on bottom). (C) Mean temperature (n = 6) and (D) and temperatures of individual animals over the same time points. (E) Mean white blood cell counts from infected baboons over the course of the experiment (see Fig 1 for number of animals included per time point). (F) Percentage of immature cells found in CBCs at day 5, and (G) levels of bacteremia, for baboons through course of experiment. Values in median and standard derivation (SD), statistical significance (*<0.05, **≤0.01, ***≤0.001, ****≤0.0001) was determined using two-tail unpaired Student’s t-test.

**Fig 3:**
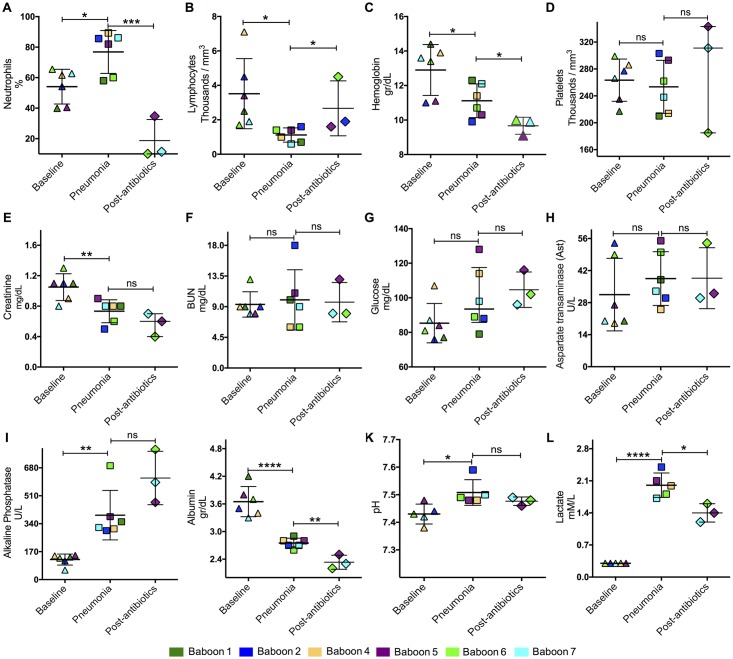
Assessment of pneumonia severity. Complete blood count (CBC) including (A) neutrophils, (B) lymphocytes, (C) hemoglobin, and (D) platelets. Renal function was assessed using (E) creatinine and (F) BUN levels. To evaluate liver function and the metabolic profile, (G) glucose, (H) aspartate transaminase, (I) alkaline phosphatase, (J) and albumin were measured. Ventilation and microcirculation was assessed by measuring (K) pH and (L) lactate. Colored symbols correspond to individual values for each baboon at day 0 (Baseline), day 4 post-infection (Pneumonia), and 5 days after antimicrobial treatment (post-antibiotics). Statistical significance (*<0.05, **≤0.01, ***≤0.001, ****≤0.0001) was determined using two-tail unpaired Student’s *t*-test.

Seven unrelated healthy baboons were enrolled in the study. Four males (57%) and three (43%) females. The median age of all baboons was 11 (IQR, 10–19) years old, which corresponds to a middle-aged to elderly human. An overview of the experiments and details of individual baboons is provided in Fig 1. Readers will note that baboon #3 developed mild pneumonia, whereas all others developed severe disease. For this reason, data from this baboon is not presented as part of Figs 2 and 3. Figures with data of the entire cohort (including baboon 3) are presented in supplemental figures (S2 and S3 Figs).

### Clinical and Hemodynamic Findings

Baboons developed dyspnea, tachypnea, and cough during the first 5 days post-inoculation when pneumonia was diagnosed (Table 1). Within 12h of intrabronchial inoculation with *S*. *pneumoniae*, animals had an increased heart rate (Fig 2A and 2B) and hyperthermia (Fig 2C and 2D). Resolution of the tachycardia and hyperthermia occurred in animals that received antibiotics (Fig 2A–2D). Leukocytosis was detected 24h post-infection in all the animals, but resolved with subsequent leukopenia after day 5 of the experiment (Fig 2E, S2 Fig). Increased immature white blood cells were seen in two animals at day 4 of the experiment when pneumonia was diagnosed (Fig 2F, S2 Fig). Bacteremia was observed in all the animals 24 h post-infection, but resolved after administration of ampicillin in all 3 baboons given antibiotics (Fig 2G, S2 Fig). When pneumonia was diagnosed on day 4 post-infection, several markers were significantly increased, including neutrophils (Fig 3A), lymphocytes (Fig 3B), alkaline phosphatase (Fig 3I), pH (Fig 3K), and lactate levels (Fig 3L). In contrast, hemoglobin (Fig 3C), creatinine (Fig 3E), and albumin (Fig 3J) levels significantly decreased by day 5 post-infection. There were no statistically significant differences between the other laboratory tests (Fig 3D and 3F–3H). Therefore, a combination of clinical signs and symptoms with corresponding abnormal laboratory tests were suggestive of systemic inflammatory response syndrome (SIRS) and history of intrabronchial bacterial inoculation confirmed a diagnosis of pneumonia.

**Table 1:** Pneumonia symptoms, ultrasound and pathology findings.

Baboon	Pneumonia symptoms					Diagnosis	
	Fever	Cough	Tachypnea	Dyspnea	Tachycardia	Ultrasound	Pathology
**1**	Yes	**No**	Yes	Yes	Yes	Multilobular consolidation	Confluent pneumonia
**2**	Yes	Yes	Yes	Yes	Yes	Multilobular consolidation	Abscessed pneumonia
**3**	Yes	**No**	**No**	**No**	Yes	1 lobe consolidation	Localized pneumonia
**4**	Yes	Yes	Yes	Yes	Yes	Multilobular consolidation	Pneumonia
**5**	Yes	Yes	Yes	**No**	Yes	Multilobular consolidation	Confluent pneumonia
**6**	Yes	Yes	Yes	Yes	Yes	Multilobular consolidation	Abscessed pneumonia
**7**	Yes	Yes	Yes	Yes	Yes	Multilobular consolidation	Confluent pneumonia

### Inflammatory Response

**Fig 4:**
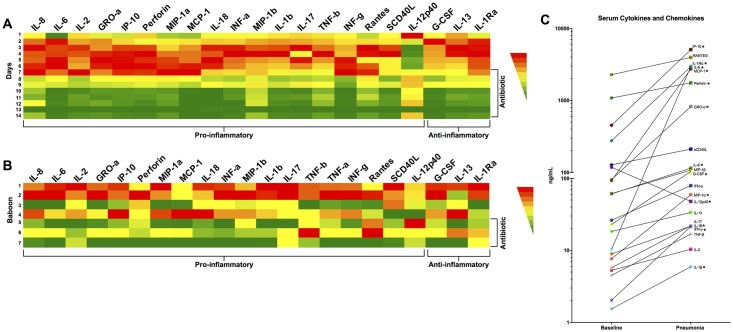
Cytokines and chemokines present in serum and lungs during pneumococcal pneumonia and following antibiotic intervention. Heat map represents the mean concentration of 20 cytokines and chemokines measured in baboons with pneumococcal pneumonia. Panel A represents mean concentration of cytokines and chemokines in serum of all animals stratified by days post challenge. Panel B show the mean lung homogenized tissue levels of 20 cytokines and chemokines measured at end of the experiment and stratified by individual animal. The color represents the concentration range from highest (red) to lowest (green) for each cytokine/chemokine. (C) Individual values for these cytokines and chemokines in serum at baseline (day 0), and during pneumonia (day 4). Statistical significance (*<0.05) was determined using paired nonparametric Mann-Whitney U tests.

We observed a progressive elevation of serum cytokine and chemokine levels including IL-6, IL-1β, IFN- α, IFN- γ, IL-17, and IL-1Ra beginning 24 h post-infection; only IL-12p40 was not elevated. Marked improvement in cytokine and chemokine levels were seen in the 3 animals that received ampicillin treatment (Fig 4A and 4B). Cytokine and chemokine levels were elevated in homogenized lung tissue samples at the end of the experiment but suppressed in baboons treated with ampicillin treatment (Fig 4C). The complete cytokine and chemokines values are presented in the supplementary material (S2 Table).

### Lung Ultrasound Findings

**Fig 5:**
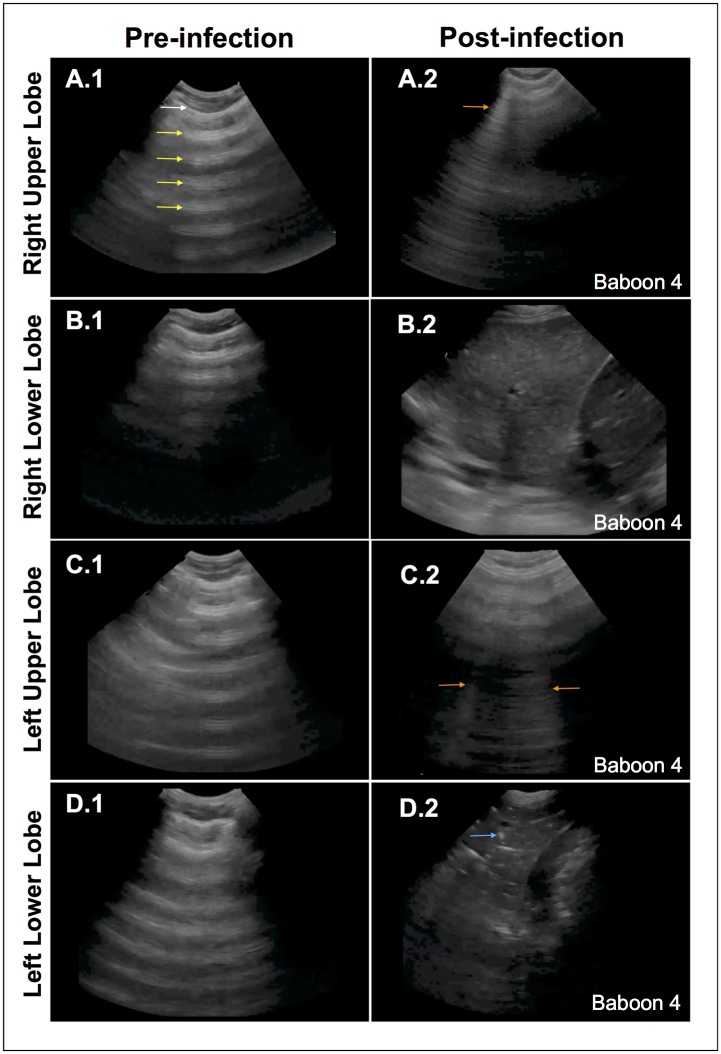
Ultrasonography diagnosis of pneumonia. Representative images of baseline ultrasonography (A.1-D.1) demonstrate normal lung findings: normal pleura (*white arrow*) with reverberations of the pleura, or A-lines, seen in the air-filled lung (*yellow arrows*). The end-of-experiment images demonstrate different pathologic findings. The right upper lobe (A2) shows a discrete, vertical hyperechoic line, or B-line (*orange arrow*), a type of reverberation artifact due to interlobular septal edema. The right lower lobe (B2) shows a densely consolidated lobe that has similar echogenicity as the liver (“hepatization”) due to replacement of lung air with fluid. The left upper lobe (C2) shows a few B-lines (*orange arrows*) due to interstitial edema. The left lower lobe (D2) shows a densely consolidated lobe with distinct white speckled areas that are air-bronchograms due to the air-water interface in the terminal bronchioles (*blue arrow*). A small pleural effusion (*) is also seen.

**Fig 6:**
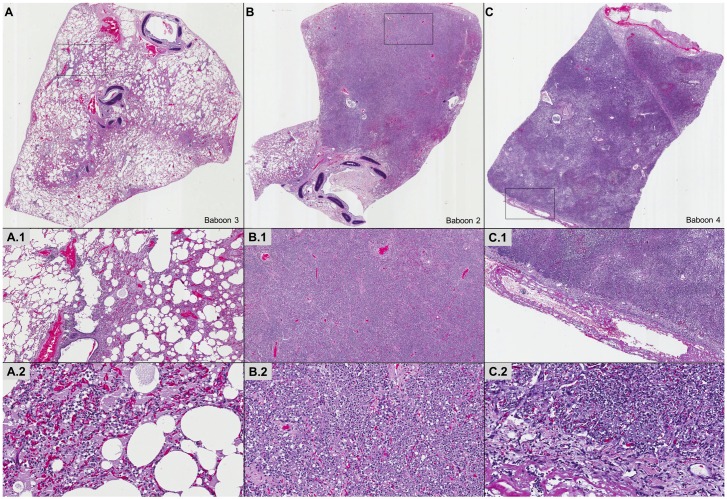
Lung infection with *S*. *pneumoniae* leads to pneumonia with variable pathological characteristics. Representative hematoxylin & eosin images of lung specimens from infected baboons. Mild (A), moderate (B), and severe (C) histopathological grades are depicted. Histopathologically, mild pneumonia was localized in and about airway and vascular sites (A). Involved bronchioles and subjacent alveoli contained edema and varying numbers of neutrophils and vascular red blood cell (RBC) congestion (A1, A2). Moderate pneumonia had intense peribronchiolar alveolar filling with fibrinopurulent exudates of neutrophils, edema, fibrin, collections of RBCs and mononuclear cells without extension throughout the entire lobe (B, B1, B2). Severe pneumonia included extensive lobar consolidating lesions of fibrinopurulent exudates, pyknotic nuclear debris, focal RBC hemorrhages, mononuclear cells and striking fibrinopurulent pleuritis (C, C1, C2).

During the baseline assessment, the lungs were examined with ultrasound to confirm a normal appearance defined by normal pleural sliding and presence of A-lines, a reverberation artifact of the pleural line (Fig 5A1–5D1). By the end of the experiment, the lung ultrasound exam revealed various abnormal findings that were based on the severity of infection. B-lines, discrete, laser-like hyperechoic lines that emanate from the pleura, were seen as interstitial fluid accumulated due to inflammation in early pneumonia (Fig 5A2 and 5C2). Dense consolidation of the lung appeared as homogenously echogenic tissue, similar in appearance to the liver (“hepatization”), due to increasing fluid content in the infected lobe (Fig 5B.2). Additionally, dynamic air-bronchograms were seen, small pockets of trapped air in the terminal bronchioles that appeared as white specks and moved with respiration (Fig 6D2). Pleural effusions were visualized as anechoic (black) areas in the costophrenic recesses (Fig 6D2). Although these findings varied depending on the severity of infection in a particular section of lung, these findings were observed in all animals and were consistent with a diagnosis of pneumonia.

### Histopathological Findings

**Fig 7:**
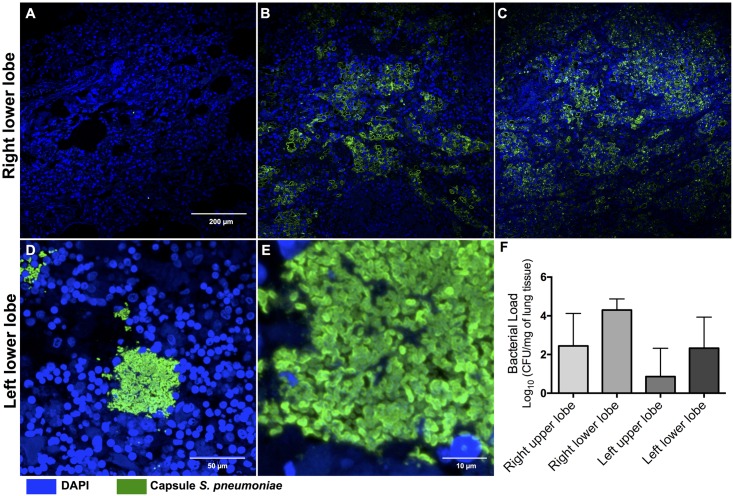
*S*. *pneumoniae* disseminates within the lungs during pneumonia. Representative images of lung sections from the 7 baboons infected with *S*. *pneumoniae*. Pneumococci were visualized using immunofluorescence by staining with antiserum against serotype 4 capsular polysaccharide (CPS) (**green**). Single pneumococci were scattered throughout the lungs even during mild-pneumonia (A), increasing in intensity and number during moderate (B) and severe pneumonia (C). High magnification images of pneumococci in the left lower lobe stained for CPS at (D) 40X and (E) 100X magnification. This revealed bacterial aggregates with diplococci morphology within the lungs. DAPI was used to reveal nucleated tissue cells for all sections. (F) Median bacterial load in each lobe of infected animals at time of euthanasia in baboons that did not receive antimicrobial therapy (n = 4).

Macroscopic examination of the lungs at the end of the experiment demonstrated evidence of lobar pneumonia with different degrees of severity (Table 1). Pleural effusions were detected in 2 baboons. Microscopic examination with H&E stained slides from 1 baboon with mild pneumonia showed small lesions localized to the airways and vasculature. The involved bronchioles and adjacent alveoli were edematous with varying numbers of neutrophils and red blood cells (RBC) due to vascular congestion (Fig 6A1 and 6A2). Two baboons developed moderate pneumonia with large areas of consolidation and extensive peribronchiolar alveolar filling with fibrinopurulent exudates containing neutrophils, fibrin, RBCs and mononuclear cells, although the entire lobe was not affected (Fig 6B1 and 6B2). Four baboons had severe pneumonia including extensive lobar consolidation with fibrinopurulent exudates, pyknotic nuclear debris, focal hemorrhages, mononuclear cells, and striking fibrinopurulent pleuritis (Fig 6C1 and 6C2). The histopathological findings in the lungs correlated with the lung ultrasound findings. Finally, we performed immunofluorescent staining of the lung parenchyma in all animals and confirmed the presence of *S*. *pneumoniae* in baboons that did not receive ampicillin (Fig 7A–7E). Fluorescent ring-like structures, suggestive of pneumococcal infiltration in the alveolar spaces, were observed in all but one lung (Fig 7D and 7E). Elevated pneumococcal bacterial loads were recovered from all affected homogenized lung sections (Fig 7F, S1 Fig).

## Discussion

The results of our study demonstrate that baboons develop pneumococcal pneumonia after intrabronchial inoculation that closely resembles infection in humans. Baboons consistently developed the classic signs and symptoms of pneumococcal pneumonia in humans, including cough, dyspnea, tachypnea, fever, tachycardia and leukocytosis [8, 9, 24]. A robust inflammatory response reflecting disease severity ensured after inoculation and improved after administration of antibiotic therapy. We demonstrated that lung ultrasonography is a remarkable tool to accurately diagnose pneumonia using *ex vivo* histopathological confirmation. Finally, we were able to isolate pneumococci from lung parenchyma and serum. Our findings are consistent with data from the pre-antibiotic era that demonstrated high mortality rates associated with invasive pneumococcal pneumonia and the post-antibiotic era with reduced mortality rates [8, 9].

In 1890, Robert Koch proposed four postulates to establish a causative relationship between pathogen and disease [25]. Koch’s postulates were met by first showing an abundance of *S*. *pneumoniae* in a human patient with pneumonia [26]. Second, the pathogen was isolated from the diseased individual and grown in pure culture (i.e. strain TIGR4). Third, TIGR4 grown in pure culture caused pneumonia when inoculated into a healthy adult laboratory baboon. Finally, the pathogen was re-isolated from baboons with pneumonia and shown to be the same as the original inoculated bacterium using immunofluorescent microscopy. Therefore, we confirmed that the pneumonia in our baboons was due to pneumococcal inoculation and the clinical, laboratory, and pathological manifestations resemble pneumococcal disease in humans.

Current rodent and small animal models are limited due to differences in anatomy, physiology, and host pathogen interaction from humans [12, 13]. Thus, development of an experimental model for pneumococcal disease in humans is a priority for future development of diagnostic, therapeutic, and preventive strategies. Non-human primates are the closest animals to humans by evolutionary proximity and other characteristics, including bipedalism, organ anatomy, physiology, and immune response to infection. These characteristics allowed us to develop a novel model reflecting the natural history of pneumococcal pneumonia in humans, managed with and without antibiotic treatment. Of note, prior attempts to develop a non-human primate model for pneumococcal pneumonia differed from ours by (1) use of more indolent pneumococcal strains that caused a subacute or mild pneumonia [27–29], (2) fragmented clinical evaluation protocols [30], and (3) diagnostic approaches centering on traditional radiography [27, 31].

In our study, we used lung ultrasonography, a novel point-of-care diagnostic modality. Lung ultrasound has several advantages over traditional radiography, including the ability to rapidly obtain several views of a particular segment or lobe of lung as well adjacent organs; it avoids transporting patients to radiographic imaging suites which is costly and inconvenient; it can differentiate lung consolidation from pleural effusion with high accuracy; and allows physicians to monitor disease states serially with repeat evaluation performed at the discretion of the investigator. Lung ultrasonography has been shown to be a superior diagnostic method compared to chest radiography, as shown in several published meta-analyses [32–34]. In our study, the in-vivo lung ultrasonography had a perfect correlation with the pathological findings identified at the time of necropsy.

We demonstrated pneumococcal invasion of the lung parenchyma using immunofluorescent microscopy of tissue sections [35]. The two phases of our experiment allowed us to compare the differences in cytokines, chemokines, and organ involvement during the systemic inflammatory response with and without antibiotic therapy [36]. All of these data suggest that the inflammatory response observed in our experimental pneumonia baboon model translates to findings observed in humans [36–39].

Our research project has some important limitations. First, the sample size was limited to 7 animals due to the high costs associated with each experimental animal. Second, we did not have the equipment to confirm hemodynamic instability during the study period. Our study was designed to mimic pneumonia in humans that may require hospitalization but not intensive care unit admission where invasive monitoring is most likely to be performed [3, 38]. Finally, changes in mental status that are commonly observed in septic humans [2, 24] could not be systematically and objectively evaluated in this non-human primate model. However, alertness and usual behavior of the animals, such as feeding and willingness to interact, was continuously monitored and recorded.

Given the similarity between non-human primates and humans we believe that one considerable strength of our model will be its use to examine the basis of sequelae that are associated with hospitalization for severe pneumococcal disease. Clinical epidemiological studies suggest that hospitalization for pneumonia is linked to new or worsened cardiac dysfunction [40], kidney failure [41], and other morbidities during convalescence that collectively contribute to an increased mortality rate for up to 10 years thereafter [42]. Since our baboons could be rescued with antimicrobials following development of severe pneumonia, these animals could be monitored following intervention and examined in detail to determine the basis for these clinical features.

In summary, we have demonstrated that the intrabronchial inoculation of *S*. *pneumoniae* in baboons can cause clinical characteristics, organ involvement, disease severity, inflammatory response and progression of the disease that closely resembles pneumococcal pneumonia in humans. Additionally, we provided evidence that lung ultrasonography is a reliable point-of-care diagnostic tool that can non-invasively detect pneumonia. Finally, this novel experimental pneumonia model could serve in future investigations to elucidate the host-pathogen interaction, test novel diagnostic methods, and evaluate new therapeutic and preventive strategies, with the ultimate goal of improving translation of new discoveries to humans.

## Supporting Information

S1 Fig Lung bacterial load per animal (n = 7). At the end of the experiment, lung parenchyma was homogenized and cultured to determine bacterial burden per lung lobes. Median bacterial load in each lobe of infected animals (n = 7).
(TIFF)

S2 Fig Animals developed systemic inflammatory response syndrome after intrabronchial challenge, entire cohort (n = 7). Animals developed systemic inflammatory response syndrome after intrabronchial challenge. Tethered animals were continuously monitored to measure heart rate and temperature. (A) Mean heart rate (n = 7) 5 days before and after intratracheal challenge with *S*. *pneumoniae*. (B) Individual heart rates for the baboons at day 0 (Baseline), day 4 post-infection (Pneumonia), and 4–5 days after antimicrobial treatment (post-antibiotics). Each different colored symbol represents an individual animal (legend on bottom). (C) Mean temperature (n = 7) and (D) and temperatures of individual animals over the same time points. (E) Mean white blood cell counts from infected baboons over the course of the experiment (see Fig 1 for number of animals included per time point). (F) Percentage of immature cells found in CBCs at day 5, and (G) levels of bacteremia, for baboons through course of experiment. Values in median and standard derivation (SD), statistical significance (*<0.05, **≤0.01, ***≤0.001) was determined using two-tail unpaired Student’s t-test.
(TIFF)

S3 Fig Assessment of pneumonia severity in the entire cohort (n = 7). Assessment of pneumonia severity. Complete blood count (CBC) including (A) neutrophils, (B) lymphocytes, (C) hemoglobin, and (D) platelets. Renal function was assessed using (E) creatinine and (F) BUN levels. To evaluate liver function and the metabolic profile, (G) glucose, (H) aspartate transaminase, (I) alkaline phosphatase, (J) and albumin were measured. Ventilation and microcirculation was assessed by measuring (K) pH and (L) lactate. Colored symbols correspond to individual values for each baboon at day 0 (Baseline), day 4 post-infection (Pneumonia), and 5 days after antimicrobial treatment (post-antibiotics). Statistical significance (*<0.05, **≤0.01, ***≤0.001) was determined using two-tail unpaired Student’s *t*-test.
(TIFF)

S1 Table List of cytokines and chemokines evaluated. (DOCX)

S2 Table Individual cytokines and chemokines concentration per animal. (DOCX)

S1 Text Supplemental material and methods. (DOCX)
