# Supplementary figures and images for: A Non-Human Primate Model of Severe Pneumococcal Pneumonia
Source: PLoS One. 2016 Nov 17;11(11):e0166092. doi: 10.1371/journal.pone.0166092 (PMC5113940; doi:10.1371/journal.pone.0166092)

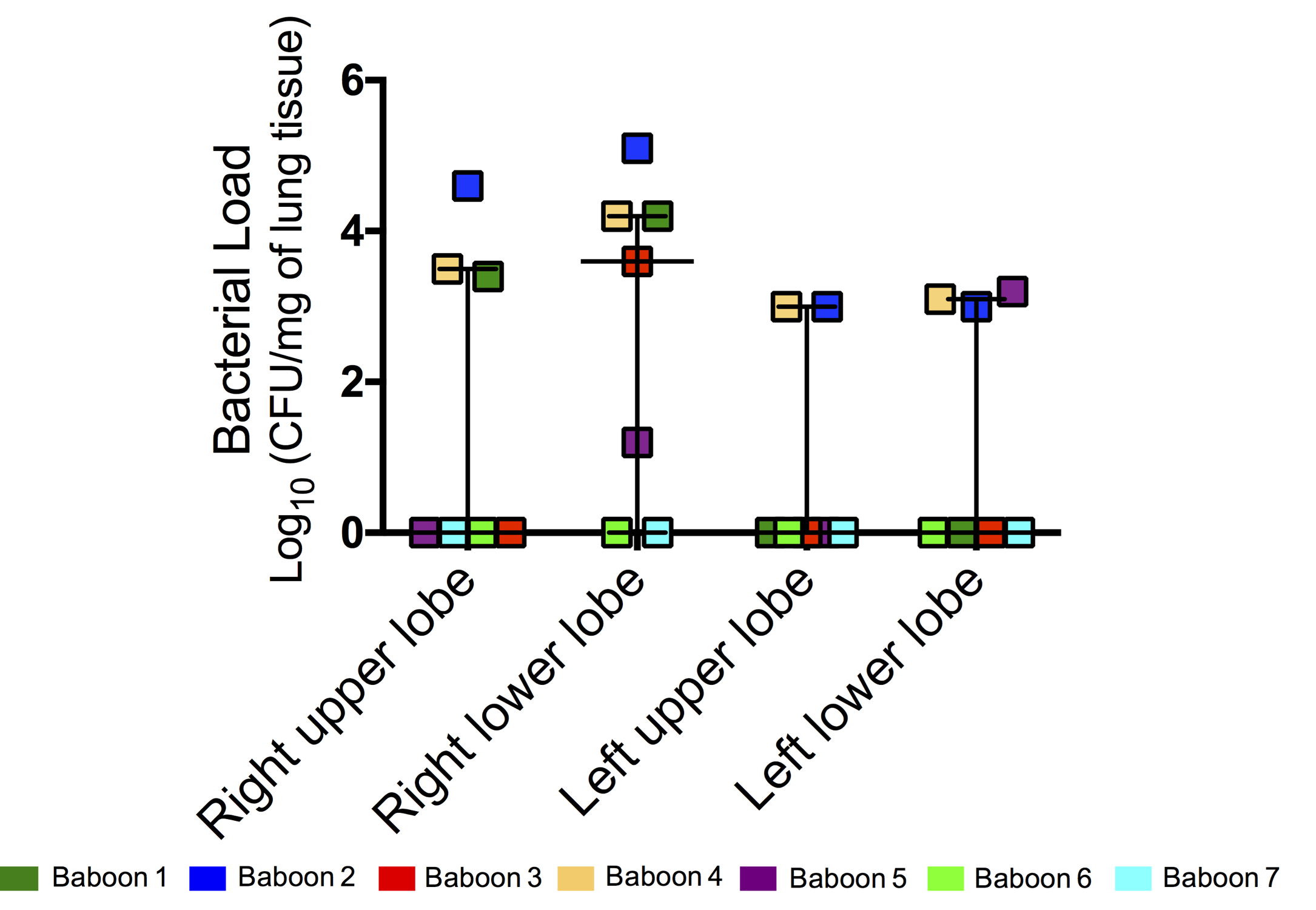

Supplement: S1 Fig — At the end of the experiment, lung parenchyma was homogenized and cultured to determine bacterial burden per lung lobes. Median bacterial load in each lobe of infected animals (n = 7). (TIFF) [file pone.0166092.s001.tiff]

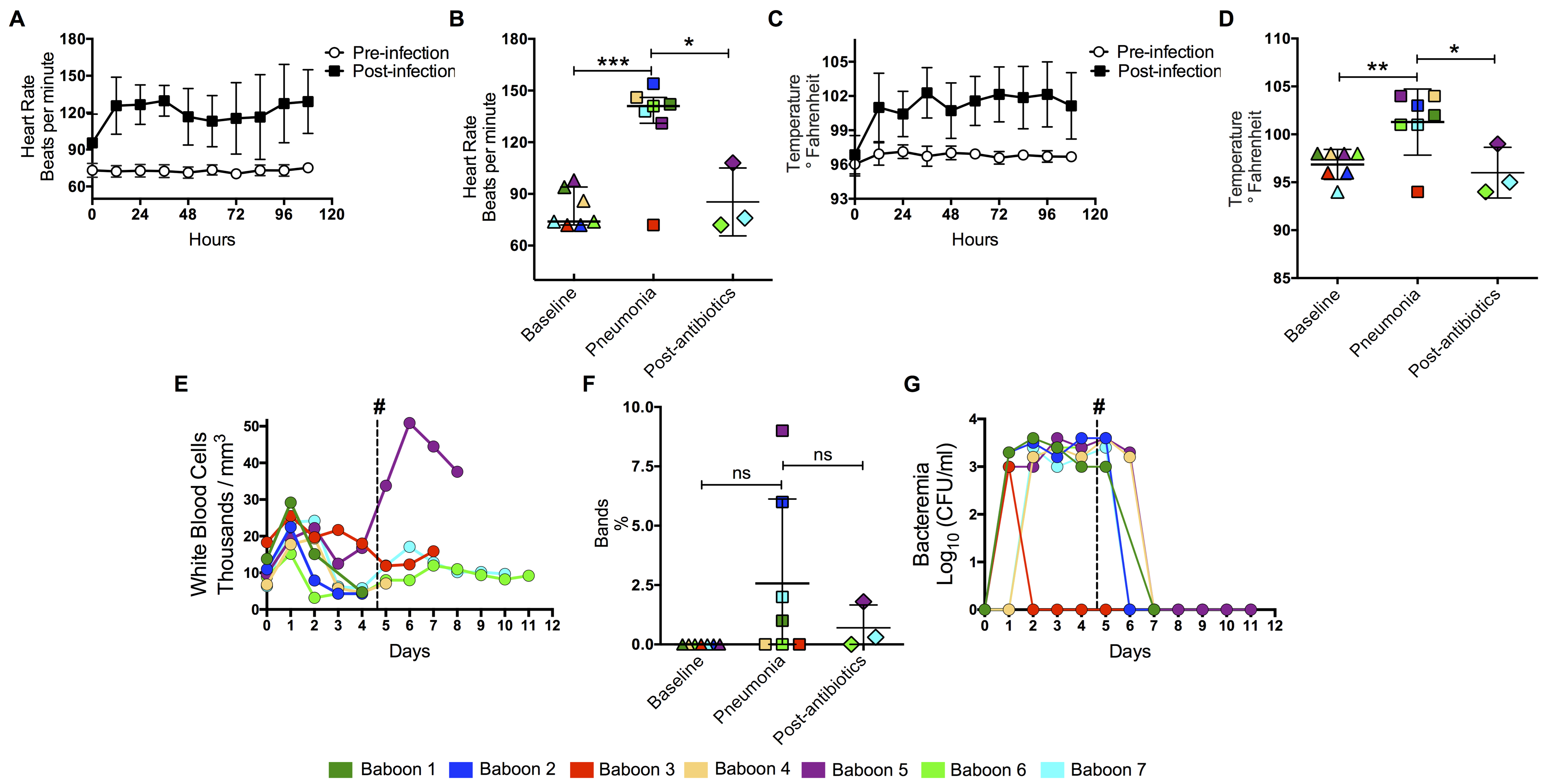

Supplement: S2 Fig — Animals developed systemic inflammatory response syndrome after intrabronchial challenge. Tethered animals were continuously monitored to measure heart rate and temperature. (A) Mean heart rate (n = 7) 5 days before and after intratracheal challenge with S. pneumoniae. (B) Individual heart rates for the baboons at day 0 (Baseline), day 4 post-infection (Pneumonia), and 4–5 days after antimicrobial treatment (post-antibiotics). Each different colored symbol represents an individual animal (legend on bottom). (C) Mean temperature (n = 7) and (D) and temperatures of individual animals over the same time points. (E) Mean white blood cell counts from infected baboons over the course of the experiment (see Fig 1 for number of animals included per time point). (F) Percentage of immature cells found in CBCs at day 5, and (G) levels of bacteremia, for baboons through course of experiment. Values in median and standard derivation (SD), statistical significance (*<0.05, **≤0.01, ***≤0.001) was determined using two-tail unpaired Student’s t-test. (TIFF) [file pone.0166092.s002.tiff]

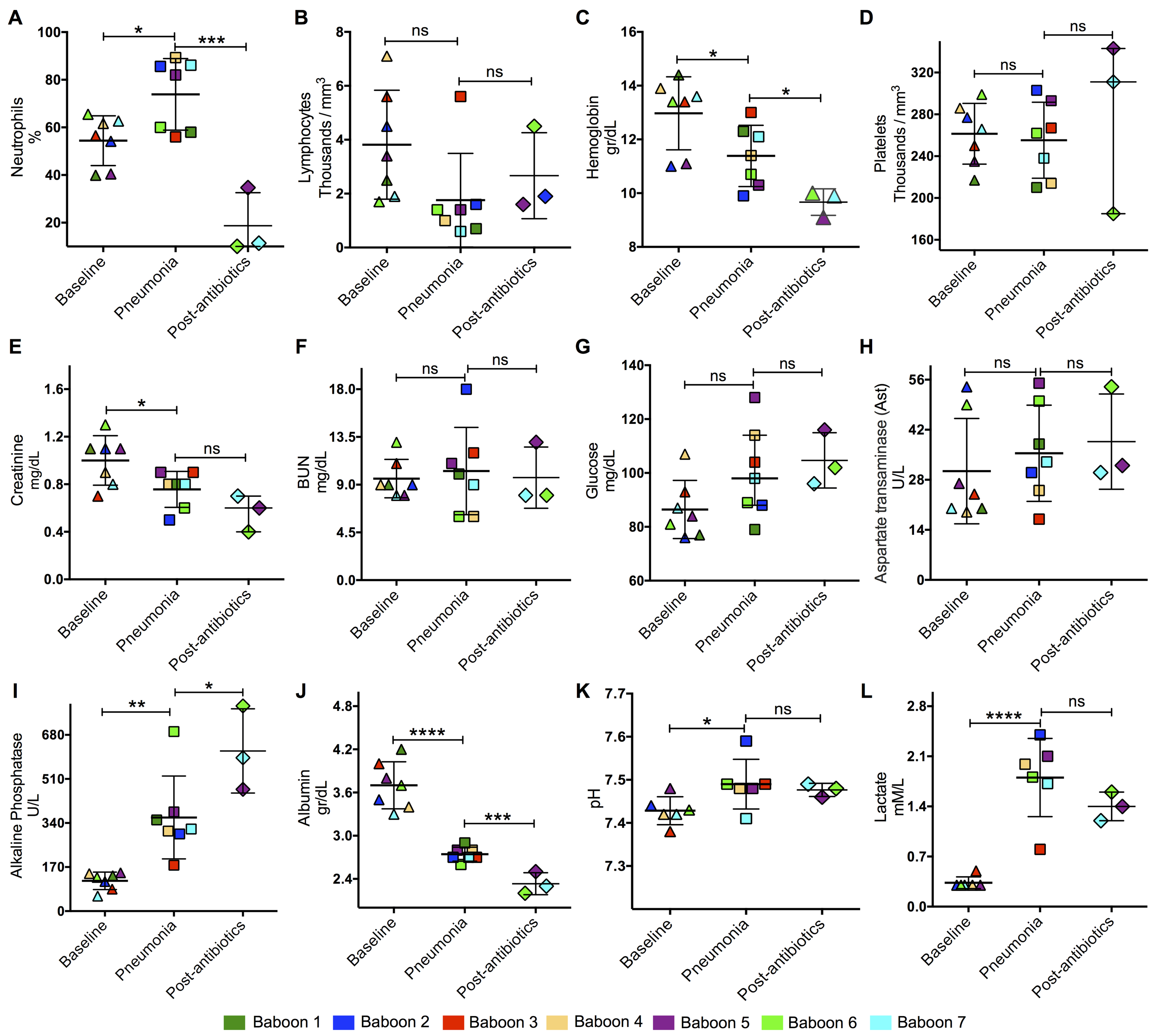

Supplement: S3 Fig — Assessment of pneumonia severity. Complete blood count (CBC) including (A) neutrophils, (B) lymphocytes, (C) hemoglobin, and (D) platelets. Renal function was assessed using (E) creatinine and (F) BUN levels. To evaluate liver function and the metabolic profile, (G) glucose, (H) aspartate transaminase, (I) alkaline phosphatase, (J) and albumin were measured. Ventilation and microcirculation was assessed by measuring (K) pH and (L) lactate. Colored symbols correspond to individual values for each baboon at day 0 (Baseline), day 4 post-infection (Pneumonia), and 5 days after antimicrobial treatment (post-antibiotics). Statistical significance (*<0.05, **≤0.01, ***≤0.001) was determined using two-tail unpaired Student’s t-test. (TIFF) [file pone.0166092.s003.tiff]
