# Supplementary material for: A Non-Human Primate Model of Severe Pneumococcal Pneumonia
Source: PLoS One. 2016 Nov 17;11(11):e0166092. doi: 10.1371/journal.pone.0166092 (PMC5113940; doi:10.1371/journal.pone.0166092)
Supplement: S1 Table — (DOCX) [file pone.0166092.s004.docx]

|  | **Short name** | **Complete name** |
| --- | --- | --- |
| 1 | IL 1-b | Interleukin 1 beta |
| 2 | G-CSF | Granulocyte-colony stimulating factor |
| 3 | GRO-a | Growth-regulated alpha protein |
| 4 | IL-2 | Interleukin 2 |
| 5 | IL-6 | Interleukin 6 |
| 6 | MIP-1a | Macrophage inflammatory protein 1-alpha |
| 7 | MIP-1b | Macrophage inflammatory protein 1-beta |
| 8 | IL-12p40 | IL - 12 subunit p40 |
| 9 | IP-10 | IFN-gamma-inducible protein 10 |
| 10 | IL - 18 | Interleukin 18 |
| 11 | IFN - a | Interferon - alpha |
| 12 | IL-13 | Interleukin 13 |
| 13 | PRF | Perforin |
| 14 | IL-17 | Interleukin 17 |
| 15 | MCP-1 | Monocyte Chemoattractant Protein-1 |
| 16 | TNF - b | Tumor necrosis factor-beta |
| 17 | IL-1Ra | Interleukin-1 receptor antagonist |
| 18 | IFN -g | Interferon - gamma |
| 19 | TNF - a | Tumor necrosis factor-alpha |
| 20 | RANTES | Regulated on activation, normal T cell expressed and secreted |
| 21 | IL-8 | Interleukin 8 |
| 22 | Scd40l | Soluble CD40 ligand |
