# Supplementary material for: A Non-Human Primate Model of Severe Pneumococcal Pneumonia
Source: PLoS One. 2016 Nov 17;11(11):e0166092. doi: 10.1371/journal.pone.0166092 (PMC5113940; doi:10.1371/journal.pone.0166092)
Supplement: S2 Table — (DOCX) [file pone.0166092.s005.docx]

| **Serum** | |  |  |  |  |  |  |  |  |  |  |  |
| --- | --- | --- | --- | --- | --- | --- | --- | --- | --- | --- | --- | --- |
| **Animal** | **Time** | **IL-1**β pg/mL | **G-CSF** pg/mL | **GRO-**α pg/mL | **IL-2** pg/mL | **IL-6** pg/mL | **MIP-1**α pg/mL | **MIP-1**β pg/mL | **IL-12p40** pg/mL | **IP-10** pg/mL | **IL-18** pg/mL | **IFN-**α pg/mL |
| **Baboon 1** | 0 h | 2.4 | 18.6 | 93.2 | 1.7 | 13.8 | 9.5 | 60.3 | 24.8 | 702.9 | 3.8 | 26.1 |
|  | 24 h | 5.7 | 594.1 | 277.5 | 4.5 | 1,568.6 | 17.0 | 70.7 | 16.8 | 1,466.2 | 7.0 | 43.9 |
|  | 48 h | 9.2 | 133.3 | 266.5 | 8.4 | 1,509.9 | 22.2 | 93.6 | 15.2 | 3,069.8 | 7.8 | 72.3 |
|  | 72 h | 23.9 | 283.0 | 834.7 | 14.7 | 1,592.1 | 71.2 | 208.3 | 20.9 | 3,578.5 | 34.1 | 167.8 |
| **Baboon 2** | 0 h | 2.7 | 13.3 | 64.7 | 1.8 | 1.3 | 5.0 | 72.0 | 26.0 | 783.9 | 1.5 | 16.3 |
|  | 24 h | 4.2 | 177.2 | 214.4 | 3.3 | 1,171.6 | 14.1 | 79.2 | 17.8 | 1,594.3 | 4.5 | 36.2 |
|  | 48 h | 5.6 | 8.3 | 274.0 | 5.0 | 809.5 | 21.9 | 141.7 | 26.4 | 3,667.1 | 2.5 | 45.6 |
|  | 72 h | 5.3 | 35.0 | 814.4 | 5.7 | 1,038.7 | 33.0 | 129.8 | 23.8 | 4,233.2 | 6.9 | 51.0 |
|  | 96 h | 4.2 | 47.8 | 846.9 | 5.4 | 657.2 | 48.3 | 92.4 | 27.1 | 5,652.3 | 10.5 | 64.1 |
|  | 120 h | 4.1 | 62.8 | 778.3 | 6.9 | 826.7 | 56.3 | 82.6 | 23.5 | 7,316.5 | 14.8 | 73.5 |
|  | 144 h | 5.0 | 102.6 | 777.7 | 9.4 | 2,398.4 | 151.1 | 90.9 | 13.9 | 8,781.1 | 20.6 | 96.1 |
| **Baboon 3** | 0 h | 4.6 | 123.7 | 106.1 | 26.7 | 13.9 | 18.7 | 228.0 | 77.6 | 434.0 | 24.3 | 115.4 |
|  | 24 h | 5.9 | 141.1 | 231.8 | 32.4 | 82.1 | 23.2 | 261.5 | 70.5 | 1,713.0 | 29.5 | 133.1 |
|  | 48 h | 10.1 | 333.3 | 337.7 | 35.5 | 66.1 | 30.0 | 238.2 | 65.3 | 724.7 | 72.0 | 199.6 |
|  | 72 h | 3.9 | 134.8 | 185.5 | 21.7 | 28.6 | 18.2 | 166.4 | 54.2 | 1,579.7 | 20.0 | 95.6 |
|  | 96 h | 10.7 | 371.3 | 495.0 | 35.6 | 55.1 | 29.3 | 186.8 | 57.7 | 1,681.7 | 78.7 | 225.8 |
|  | 120 h | 6.3 | 277.5 | 163.7 | 29.2 | 12.6 | 23.1 | 151.4 | 50.8 | 1,557.1 | 58.5 | 194.1 |
|  | 144 h | 2.7 | 22.4 | 113.2 | 13.8 | 11.4 | 15.1 | 121.3 | 56.6 | 768.8 | 4.7 | 41.1 |
|  | 168 h | 2.7 | 61.9 | 82.6 | 13.3 | 9.5 | 14.8 | 115.9 | 53.8 | 502.7 | 9.6 | 67.0 |
|  | 192 h | 4.7 | 144.2 | 131.8 | 15.0 | 9.4 | 16.9 | 106.0 | 48.5 | 320.9 | 25.0 | 110.2 |
| **Baboon 4** | 0 h | 0.2 | <1.20 | 126.8 | <1.19 | 5.0 | 8.1 | <4.88 | 111.8 | 401.0 | 0.6 | <3.27 |
|  | 24 h | 0.4 | 8.9 | 202.2 | 1.9 | 2,311.7 | 16.1 | <4.88 | 38.5 | 636.1 | 0.6 | <3.27 |
|  | 48 h | 0.4 | 6.3 | 160.8 | 5.9 | 1,176.1 | 19.7 | <4.88 | 34.3 | 4,192.9 | 0.7 | 54.9 |
|  | 72 h | 0.6 | 17.7 | 519.7 | 13.7 | 2,979.6 | 51.3 | 9.9 | 38.8 | 4,863.7 | 0.7 | 69.0 |
|  | 96 h | 1.0 | 10.9 | 916.4 | 10.2 | 6,033.0 | 140.1 | 57.2 | 49.7 | 9,872.7 | 0.7 | 41.8 |
|  | 120 h | 0.6 | 3.5 | 754.8 | 3.6 | 4,943.4 | 146.4 | <4.88 | 32.5 | 6,909.0 | 0.7 | 22.0 |
|  | 144 h | 0.6 | 6.6 | 1,097.2 | 67.1 | 6,281.9 | 151.0 | 72.3 | 37.0 | 10,103.9 | 0.7 | 22.0 |

| **Serum** | |  |  |  |  |  |  |  |  |  |  |  |
| --- | --- | --- | --- | --- | --- | --- | --- | --- | --- | --- | --- | --- |
| **Animal** | **Time** | **IL-1**β pg/mL | **G-CSF** pg/mL | **GRO-**α pg/mL | **IL-2** pg/mL | **IL-6** pg/mL | **MIP-1**α pg/mL | **MIP-1**β pg/mL | **IL-12p40** pg/mL | **IP-10** pg/mL | **IL-18** pg/mL | **IFN-**α pg/mL |
| **Baboon 5** | 0 h | 0.2 | <1.20 | 121.0 | 2.7 | 10.7 | 7.8 | <4.88 | 168.7 | 228.9 | 0.6 | <3.27 |
|  | 24 h | 0.3 | 38.3 | 616.4 | <1.19 | 3,396.1 | 14.8 | <4.88 | 55.6 | 997.1 | 0.6 | <3.27 |
|  | 48 h | 0.3 | 31.5 | 292.5 | 20.7 | 1,331.2 | 17.5 | 15.7 | 52.3 | 2,345.0 | 0.8 | 73.8 |
|  | 72 h | 0.3 | 11.8 | 134.0 | 13.3 | 1,062.2 | 15.5 | <4.88 | 45.8 | 3,598.6 | 0.7 | 47.2 |
|  | 96 h | 0.6 | <1.20 | 528.2 | 2.7 | 1,054.8 | 15.4 | <4.88 | 54.6 | 2,670.2 | 0.7 | <3.27 |
|  | 120 h | 0.8 | 2.7 | 360.6 | 5.2 | 2,475.7 | 16.3 | 41.8 | 54.6 | 1,403.5 | 0.7 | <3.27 |
|  | 144 h | 0.8 | 6.0 | 166.0 | 5.1 | 189.4 | 18.9 | 126.8 | 77.8 | 701.5 | 0.7 | 25.1 |
|  | 168 h | 1.0 | 4.4 | 170.9 | 3.5 | 86.9 | 19.1 | 184.6 | 150.6 | 939.9 | 0.6 | 22.0 |
|  | 192 h | 1.1 | 1.9 | 107.3 | 3.6 | 46.7 | 19.6 | 289.1 | 159.9 | 665.4 | 0.6 | 22.0 |
|  | 216 h | 0.9 | 4.4 | 146.9 | 2.7 | 28.9 | 16.7 | 312.5 | 167.4 | 539.2 | 0.6 | 29.8 |
|  | 240 h | 1.6 | 8.5 | 118.1 | 7.4 | 22.4 | 25.3 | 326.9 | 167.4 | 546.3 | 0.7 | 27.6 |
|  | 264 h | 1.0 | 5.2 | 240.0 | 6.7 | 42.4 | 20.6 | 350.8 | 201.4 | 583.2 | 0.6 | <3.27 |
|  | 312 h | 0.8 | 2.7 | 131.0 | 3.9 | 23.4 | 17.4 | 236.8 | 240.7 | 390.6 | 0.6 | <3.27 |
| **Baboon 6** | 0 h | 1 | 2 | 131 | 3 | 8 | <2.19 | 33 | 318 | 441 | 5 | 10 |
|  | 24 h | 1 | 18 | 934 | 8 | 5,527 | <2.19 | 58 | 92 | 788 | 6 | 10 |
|  | 48 h | 1 | 5 | 1,123 | 8 | 5,886 | <2.19 | 70 | 59 | 8,020 | 17 | 59 |
|  | 72 h | 1 | 5 | 1,834 | 5 | 4,761 | 36 | 106 | 66 | 9,845 | 13 | 92 |
|  | 96 h | 1 | 4 | 1,960 | 4 | 4,889 | 60 | 102 | 83 | 8,699 | 12 | 38 |
|  | 120 h | 1 | 4 | 2,398 | 3 | 6,947 | 85 | 118 | 88 | 11,523 | 15 | 47 |
|  | 144 h | 0 | 3 | 539 | <0.27 | 1,633 | 77 | 75 | 86 | 7,774 | 16 | 30 |
|  | 168 h | 0 | 2 | 485 | 0 | 605 | 68 | 58 | 115 | 2,065 | 8 | <3.27 |
|  | 192 h | 1 | 2 | 290 | <0.27 | 490 | 33 | 36 | 94 | 1,500 | 12 | 19 |
| **Baboon 7** | 0 h | 0 | 0 | 30 | <0.27 | 19 | <2.19 | 28 | 100 | 179 | 5 | 10 |
|  | 24 h | 1 | 126 | 99 | <0.27 | 4,438 | 20 | 46 | 58 | 1,068 | 9 | 10 |
|  | 48 h | 1 | 1 | 52 | <0.27 | 3,368 | 33 | 36 | 38 | 3,562 | 12 | 19 |
|  | 72 h | 1 | 2 | 82 | <0.27 | 3,224 | 45 | 51 | 47 | 3,497 | 18 | 28 |
|  | 96 h | 1 | 3 | 189 | <0.27 | 6,280 | 52 | 102 | 42 | 3,405 | 17 | 14 |
|  | 120 h | 1 | 2 | 104 | 2 | 3,955 | 41 | 111 | 60 | 4,287 | 22 | 14 |
|  | 144 h | 0 | <0.11 | 43 | <0.27 | 486 | 15 | 19 | 90 | 835 | 13 | <3.27 |
|  | 168 h | 0 | 0 | 89 | <0.27 | 110 | <2.19 | 43 | 166 | 970 | 12 | 12 |
|  | 192 h | 0 | 0 | 64 | <0.27 | 28 | <2.19 | 39 | 174 | 825 | 6 | <3.27 |
|  | 216 h | <0.01 | 0 | 61 | <0.27 | 16 | <2.19 | 9 | 199 | 667 | <2.67 | <3.27 |
|  | 240 h | <0.01 | 1 | 41 | 0 | 22 | <2.19 | 25 | 222 | 661 | <2.67 | 10 |
|  | 264 h | 0 | 0 | 53 | <0.27 | 26 | <2.19 | 16 | 268 | 598 | <2.67 | <3.27 |
|  | 288 h | 0 | <0.11 | 66 | <0.27 | 26 | <2.19 | 10 | 287 | 572 | 5 | 10 |
|  | 312 h | 0 | 0 | 62 | <0.27 | 20 | <2.19 | 15 | 223 | 463 | <2.67 | 10 |

| **Serum** | |  |  |  |  |  |  |  |  |  |  |  |
| --- | --- | --- | --- | --- | --- | --- | --- | --- | --- | --- | --- | --- |
| **Animal** | **Time** | **IL-13** pg/mL | **Perforin** pg/mL | **IL-17** pg/mL | **MCP-1** pg/mL | **TNF-**β pg/mL | **IL-1R**α pg/mL | **IFN-**γ pg/mL | **TNF-**α pg/mL | **RANTES** pg/mL | **IL-8** pg/mL | **sCD40L** pg/mL |
| **Baboon 1** | 0 h | 28.6 | 616.6 | 3.8 | 256.7 | 6.6 | 341.4 | <1.19 | <6.52 | 1,706.8 | 36.6 | 152.1 |
|  | 24 h | 38.5 | 527.7 | 6.4 | 1,125.2 | 13.5 | 4,579.4 | 2.9 | <6.52 | 4,618.7 | 120.7 | 403.3 |
|  | 48 h | 46.7 | 1,030.7 | 8.8 | 3,384.0 | 16.6 | 4,529.3 | 4.7 | <6.52 | 4,000.6 | 81.8 | 165.7 |
|  | 72 h | 117.1 | 1,355.3 | 19.8 | 4,656.8 | 64.1 | 5,246.2 | 6.2 | <6.52 | 5,646.0 | 98.3 | 415.0 |
| **Baboon 2** | 0 h | 4.8 | 991.2 | <1.22 | 84.1 | 7.2 | 322.2 | 8.7 | <0.49 | 840.4 | 66.9 | 175.2 |
|  | 24 h | 10.9 | 836.6 | <1.22 | 887.3 | 6.0 | 2,831.7 | 8.1 | <0.49 | 1,736.2 | 121.9 | 296.9 |
|  | 48 h | 16.0 | 1,341.1 | <1.22 | 3,001.4 | 11.7 | 2,523.4 | 25.3 | <0.49 | 571.0 | 107.5 | 122.4 |
|  | 72 h | 16.0 | 1,594.8 | <1.22 | 4,341.6 | 12.8 | 2,387.0 | 12.8 | <0.49 | 1,654.7 | 101.8 | 150.2 |
|  | 96 h | 17.2 | 1,938.9 | 8.5 | 8,172.5 | 9.6 | 2,943.2 | 49.8 | <0.49 | 2,776.8 | 160.1 | 166.7 |
|  | 120 h | 13.6 | 2,264.8 | 11.3 | 18,626.2 | 6.5 | 2,799.0 | 72.5 | <0.49 | 3,826.1 | 219.6 | 336.4 |
|  | 144 h | 13.3 | 2,855.3 | 14.8 | >22073.54 | 7.5 | 3,122.9 | 57.6 | <0.49 | 3,357.1 | 290.1 | 596.7 |
| **Baboon 3** | 0 h | 87.5 | 1,972.7 | 44.7 | 109.7 | 15.6 | 301.2 | 3.9 | <0.49 | 2,400.2 | 131.3 | 437.6 |
|  | 24 h | 92.5 | 1,929.0 | 57.5 | 464.1 | 19.3 | 1,701.5 | 11.6 | <0.49 | 2,167.7 | 112.3 | 246.8 |
|  | 48 h | 132.5 | 2,499.0 | 114.3 | 398.8 | 33.8 | 1,569.7 | 11.5 | <0.49 | 2,369.6 | 250.9 | 3,162.7 |
|  | 72 h | 54.1 | 2,321.1 | 18.1 | 265.5 | 15.9 | 2,133.0 | 13.3 | <0.49 | 2,213.0 | 132.3 | 3,805.0 |
|  | 96 h | 90.6 | 2,616.2 | 110.6 | 454.8 | 40.1 | 1,319.7 | 17.2 | <0.49 | 2,554.5 | 163.2 | 774.9 |
|  | 120 h | 70.9 | 2,393.4 | 64.9 | 257.2 | 23.0 | 614.8 | 8.0 | <0.49 | 2,176.8 | 171.3 | 617.6 |
|  | 144 h | 46.7 | 1,679.5 | 9.6 | 141.0 | 8.4 | 256.6 | 3.7 | <0.49 | 1,550.7 | 100.0 | 338.5 |
|  | 168 h | 49.4 | 1,616.0 | 14.5 | 142.7 | 9.6 | 267.9 | 3.5 | <0.49 | 2,122.3 | 85.5 | 244.6 |
|  | 192 h | 58.7 | 1,631.7 | 44.6 | 100.6 | 14.9 | 206.6 | 2.6 | <0.49 | 2,325.6 | 182.5 | 590.2 |
| **Baboon 4** | 0 h | 1.6 | 576.9 | <3.24 | 35.5 | <0.37 | 195.4 | <0.04 | <0.45 | 8,798.1 | 126.8 | 62.4 |
|  | 24 h | 3.9 | 500.1 | <3.24 | 255.4 | <0.37 | 2,363.5 | 0.2 | <0.45 | 6,590.4 | 105.1 | 35.3 |
|  | 48 h | 2.9 | 942.5 | <3.24 | 1,532.0 | <0.37 | 3,168.1 | 2.8 | <0.45 | 7,038.6 | 102.0 | 168.2 |
|  | 72 h | 4.3 | 1,115.8 | <3.24 | 1,444.4 | <0.37 | 3,269.9 | 3.3 | <0.45 | 9,106.3 | 183.1 | 604.2 |
|  | 96 h | 2.4 | 1,395.8 | <3.24 | 2,131.1 | <0.37 | 3,840.4 | 60.6 | <0.45 | 8,054.6 | 214.7 | 60.6 |
|  | 120 h | 0.2 | 1,208.7 | <3.24 | 1,668.0 | <0.37 | 3,648.9 | 36.2 | <0.45 | 7,226.6 | 127.5 | 48.6 |
|  | 144 h | 0.2 | 1,163.6 | <3.24 | 1,836.2 | <0.37 | 3,711.4 | 86.5 | 0.5 | 8,159.6 | 177.7 | 29.0 |

| **Serum** | |  |  |  |  |  |  |  |  |  |  |  |
| --- | --- | --- | --- | --- | --- | --- | --- | --- | --- | --- | --- | --- |
| **Animal** | **Time** | **IL-13** pg/mL | **Perforin** pg/mL | **IL-17** pg/mL | **MCP-1** pg/mL | **TNF-**β pg/mL | **IL-1R**α pg/mL | **IFN-**γ pg/mL | **TNF-**α pg/mL | **RANTES** pg/mL | **IL-8** pg/mL | **sCD40L** pg/mL |
| **Baboon 5** | 0 h | <0.24 | 839.7 | <3.24 | 46.2 | <0.37 | 358.8 | <0.04 | <0.45 | 282.2 | 35.8 | 19.8 |
|  | 24 h | <0.24 | 707.7 | <3.24 | 196.0 | <0.37 | 3,145.9 | 0.5 | <0.45 | 1,104.0 | 97.9 | 40.3 |
|  | 48 h | 0.2 | 1,205.3 | <3.24 | 613.4 | 0.4 | 3,308.0 | 1.5 | <0.45 | 9,197.0 | 134.1 | 187.9 |
|  | 72 h | 2.9 | 1,054.5 | <3.24 | 488.6 | <0.37 | 3,003.4 | 3.3 | <0.45 | 8,026.6 | 88.4 | 109.1 |
|  | 96 h | 2.5 | 1,181.8 | <3.24 | 349.8 | <0.37 | 2,326.5 | 3.5 | <0.45 | 4,149.7 | 71.6 | 28.4 |
|  | 120 h | 9.4 | 1,009.5 | <3.24 | 238.5 | 1.5 | 3,050.6 | 1.1 | <0.45 | 8,478.0 | 129.7 | 16.1 |
|  | 144 h | 20.1 | 839.1 | 9.7 | 57.8 | 3.2 | 969.8 | 0.0 | <0.45 | 10,943.2 | 237.2 | 71.2 |
|  | 168 h | 20.9 | 792.4 | 9.7 | 36.7 | 4.6 | 379.4 | <0.04 | <0.45 | 10,300.2 | 158.1 | 29.1 |
|  | 192 h | 21.7 | 750.3 | <3.24 | 23.2 | 7.6 | 370.3 | <0.04 | 1.2 | 10,702.0 | 110.3 | 28.4 |
|  | 216 h | 14.3 | 653.6 | <3.24 | 14.4 | 10.4 | 367.6 | <0.04 | 0.8 | 10,216.7 | 241.5 | 79.5 |
|  | 240 h | 28.0 | 592.3 | 9.7 | 10.6 | 15.3 | 430.3 | 0.3 | 3.7 | 12,803.5 | 176.3 | 140.2 |
|  | 264 h | 15.6 | 634.3 | <3.24 | 12.8 | 11.5 | 462.6 | 0.3 | 1.5 | 7,786.8 | 380.7 | 173.7 |
|  | 312 h | 12.0 | 491.7 | <3.24 | 9.6 | 6.6 | 399.9 | 0.3 | 3.2 | 4,805.9 | 214.7 | 75.2 |
| **Baboon 6** | 0 h | <2.67 | 1,817 | <3.18 | 75 | <0.47 | 176 | <0.17 | <0.72 | 449 | 19 | 10 |
|  | 24 h | <2.67 | 1,274 | <3.18 | 310 | 1 | 2,516 | 2 | <0.72 | 3,776 | 87 | 55 |
|  | 48 h | <2.67 | 2,260 | <3.18 | 1,595 | <0.47 | 2,851 | 7 | <0.72 | 5,792 | 165 | 215 |
|  | 72 h | <2.67 | 2,679 | <3.18 | 2,483 | 1 | 2,691 | 5 | <0.72 | 1,290 | 61 | 31 |
|  | 96 h | <2.67 | 2,289 | <3.18 | 2,029 | 1 | 2,503 | 12 | 1 | 2,461 | 67 | 13 |
|  | 120 h | <2.67 | 2,674 | <3.18 | 2,092 | <0.47 | 2,856 | 16 | 1 | 3,582 | 156 | 21 |
|  | 144 h | <2.67 | 2,310 | <3.18 | 1,929 | 1 | 1,968 | 2 | <0.72 | 4,091 | 62 | 15 |
|  | 168 h | <2.67 | 2,253 | <3.18 | 1,172 | <0.47 | 956 | 0 | <0.72 | 4,252 | 62 | 21 |
|  | 192 h | 5 | 1,951 | <3.18 | 628 | 1 | 1,537 | <0.17 | <0.72 | 4,654 | 64 | 43 |
| **Baboon 7** | 0 h | <2.67 | 760 | <3.18 | 46 | 1 | 234 | <0.17 | <0.72 | 1,459 | 10 | 33 |
|  | 24 h | <2.67 | 646 | <3.18 | 600 | 2 | 2,217 | 0 | <0.72 | 2,871 | 37 | 52 |
|  | 48 h | <2.67 | 1,179 | <3.18 | 1,229 | 1 | 2,209 | 1 | <0.72 | 2,621 | 37 | 81 |
|  | 72 h | <2.67 | 1,679 | <3.18 | 1,057 | 2 | 2,329 | 2 | <0.72 | 3,142 | 61 | 112 |
|  | 96 h | <2.67 | 1,506 | <3.18 | 1,412 | 3 | 2,514 | 1 | <0.72 | 2,060 | 28 | 18 |
|  | 120 h | 5 | 2,155 | <3.18 | 1,613 | 3 | 2,148 | 1 | <0.72 | 2,794 | 24 | 18 |
|  | 144 h | <2.67 | 1,946 | <3.18 | 208 | 2 | 1,087 | 0 | <0.72 | 2,375 | 19 | 17 |
|  | 168 h | <2.67 | 2,185 | <3.18 | 196 | 2 | 383 | <0.17 | <0.72 | 5,577 | 16 | 50 |
|  | 192 h | <2.67 | 1,671 | <3.18 | 82 | 1 | 198 | <0.17 | <0.72 | 3,255 | 27 | 34 |
|  | 216 h | <2.67 | 1,509 | <3.18 | 73 | <0.47 | 154 | <0.17 | <0.72 | 1,102 | 12 | 16 |
|  | 240 h | <2.67 | 1,281 | <3.18 | 53 | <0.47 | 252 | <0.17 | <0.72 | 3,330 | 18 | 58 |
|  | 264 h | <2.67 | 676 | <3.18 | 59 | 1 | 157 | <0.17 | <0.72 | 1,519 | 22 | 19 |
|  | 288 h | <2.67 | 779 | <3.18 | 68 | 2 | 181 | <0.17 | <0.72 | 1,730 | 41 | 40 |
|  | 312 h | <2.67 | 683 | <3.18 | 58 | 1 | 163 | <0.17 | <0.72 | 2,056 | 34 | 41 |
