# Supplementary material for: A Non-Human Primate Model of Severe Pneumococcal Pneumonia
Source: PLoS One. 2016 Nov 17;11(11):e0166092. doi: 10.1371/journal.pone.0166092 (PMC5113940; doi:10.1371/journal.pone.0166092)
Supplement: S1 Text — (DOCX) [file pone.0166092.s006.docx]

**Title:** A Non-Human Primate Model of Severe Pneumococcal Pneumonia

**Authors:** Luis F. Reyes,^1,2^ Marcos I. Restrepo,^1,2^ Cecilia A. Hinojosa,^1,2^ Nilam J. Soni,^1,2^ Anukul T. Shenoy,^4^ Ryan P. Gilley,^1,3^ Norberto Gonzalez-Jarbe,^4^ Julio R. Noda,^1,2^ Vicki T. Winter,^1^ Melisa A. de la Garza,^5^ Robert Shade,^5^ Jackie Coalson,^1^ Luis Giavedoni,^5^ Antonio Anzueto,^1,2^ and Carlos J. Orihuela^3,4^

**Supporting information**

**Supplemental material and methods**

**Implantation of the continued monitoring system**

Animals were anesthetized with 10mg/kg of intramuscular (IM) ketamine. They were prepped for surgery, including intravenous (IV) catheterization and placement of endotracheal tube. For the duration of the procedure, they were maintained at 1-3% inhaled isoflourane. Using aseptic technique, a catheter was surgically implanted in the (right or left) femoral vein. Once patency was established, the catheter was secured in place using 2-0 Ethibond**^®^** suture. The exterior length of catheter was tunneled to a predetermined site on the dorsum using a trochar, so that it could be connected to the tether system for infusion of intravenous fluids and collection of blood samples. We also implanted four 22 gauge silver wire ECG leads subcutaneously on the dorsum, one at the level of each scapula and one at the level of each wing of the ilium. ECG leads were tunneled and exited through the same predetermined site as the venous catheter in the center of the dorsum. Finally, we placed a thermistor for body temperature readings subcutaneously in the right ventral abdomen on the rostral side which was also tunneled to the same exit site on the dorsum. The ECG leads and thermistor were secured in place using 2-0 Ethibond**^®^** suture. All exit sites were closed using 2-0 Vicryl**^®^** suture. Instrumentation exiting the animal was collected in a backpack built in a vest that was tunneled through a flexible coil so that all necessary monitoring could occur. Animals were allowed to recover for 2 weeks prior to pneumococcal challenge.

**Intrabronchial challenge**

Animals were chemically suppressed with 5-7mg/kg Telazol**^®^** intramuscularly. Once adequate sedation was achieved, the laryngeal folds were locally desensitized with Cetacaine**^®^** spray. A bronchoscope was advanced to the level of the carina, where 1ml of lidocaine was administered topically. The scope was then advanced until it was wedged in lung tissue. The anatomy of the lungs naturally presented the right middle lung lobe directly in the path of the advancing bronchoscope in most animals, and therefore the right middle lobe became our target instillation site. A baseline bronchoalveolar lavage (BAL) was preformed using 20ml sterile saline, and then 10^9^ CFU of *S. pneumoniae* was instilled at the site. The bronchoscope was removed, the animal was returned to his cage, and he was observed closely until fully recovered from anesthesia.

**Lung Ultrasound Examination**

A point-of-care lung ultrasound examination to screen all baboons was performed prior to study enrollment, and pre- and post-infection lung ultrasound exams were performed during the study.  All images were acquired using a portable ultrasound machine (General Electric Logiq E Vet) equipped with a microconvex (GE model 8C-RS, 4.0-10.0 MHz) and phased-array transducer (GE model 3S-RS, 1.7-4.0 MHz).  Prior to starting each lung ultrasound exam, the subject’s medical record number, date of birth, age, weight, and height were entered in the ultrasound machine.  Clippers were used to shave the fur from the anterior and lateral chest wall prior to each examination. The machine was preset to an abdominal exam setting with a depth of 6-10cm.  Each lung was interrogated with ultrasound at 4 sites using the 8-point lung ultrasound exam per the BLUE protocol. Point 1 is located on the mid-clavicular line at intercostal space (ICS) 2-3.  Point 2 is located on the anterior axillary line at ICS 4-5.  Point 3 is located on the mid-axillary line at the level of the diaphragm.  Point 4 is located on the posterior axillary line, or the posterior most point, at the level of the diaphragm.  Point 1 evaluates the upper lobe, while points 3 and 4 evaluate the lower lobes and pleural space.  Point 2 generally evaluates the right middle lobe or left lingular lobe, although portions of the upper and lower lobes are often visualized depending on transducer position.  The lung ultrasound exam was performed with the transducer in a longitudinal orientation with the notch pointed cephalad.  Dynamic image recording with a 4-6 second video was captured at each of the 4 lung exam points bilaterally.  Images were stored locally on the ultrasound machine and then archived in a central picture archiving and communication system (PACS).

**Assessment of bacterial burden in serum and lungs**

Bacteremia was assessed over the course of the study by collection of blood every 24hrs post-infection. CFU/ml was determined by serial dilution of the blood, plating on blood agar plates, and incubation at 37°C with 5% CO2 for 16-18hours. Homogenizing pre-weighed tissues, plating serial dilutions, and incubating them for 16-18hours determined bacterial burden in lungs. Colony counts were used to determine CFU/mg.

**Pathology**

Multiple lung tissue specimens were collected, fixed with 10% neutral buffered formalin and processed in paraffin. Sections from paraffin blocks were cut at 4μm and stained with hematoxylin and eosin (H&E). All slides were scanned with the Aperio Scanscope XT (Aperio, Vista CA) and digital images of the entire tissue specimen were created.

**Immunofluorescence staining**

Frozen lung sections were prepared by embedding tissues in Optimal Cutting Temperature compound (Tissue TEK**^®^**). Sections (7μm) were cut and fixed in 10% neutral buffered formalin, washed twice with phosphate-buffered saline (PBS) for 5 minutes followed by permeabilization using sterile PBS containing 0.2% Triton X. Sections were blocked for 1 hour at 25°C with sterile PBS containing 5% goat serum. The primary antibody used was rabbit anti-serotype 4 capsular polysaccharide antibody (Statens serum Institut) diluted at 1:500 in the blocking solution and incubated over the tissue sections at 4°C for overnight. After incubation, sections were washed with 0.2%Triton X in PBS and sterile PBS. The secondary antibody used was FITC labeled goat α-rabbit antibody (Jackson Immuno Research), diluted at 1:1000 in blocking solution and incubated over sections at 25°C for 1 hour. Slides were then washed and mounted with ProLong Gold Antifade reagent containing DAPI (4=,6=-diamidino-2-phenylindole) (Life Technologies).
